# Cruzar el Atlántico: el control a distancia y la navegación ibero-atlántica en el siglo XVI

**DOI:** 10.1590/S0104-59702023000100014

**Published:** 2023-04-03

**Authors:** Santiago Guzman

**Affiliations:** i Science and Technology Studies/University College London. Londres – Reino Unido. santiago.gamez.15@ucl.ac.uk

La era de los descubrimientos supusieron una transformación social y cultural del continente europeo, relacionada con la aparición de nuevos actores y su geografía. América, y su relación con Europa, produjo cambios estructurales. Dentro de este proceso, el conocimiento producido en el siglo XVI por cosmógrafos, pilotos, cartógrafos y cronistas fue fundamental para consolidar del proyecto imperial español sobre el Nuevo Mundo*.* El desafío más grande de tal expansión imperial y religiosa fue el problema de “controlar a distancia”. Este, que es esencialmente un problema de comunicación, no se puede pensar desde la historia de la ciencia sin referirse a la relación entre religión, exploración e imperio. Mauricio Nieto (2022) nos relata el proceso de conquista y expansión española como una empresa enfocada en la identificación y el control de una ruta para realizar el cruce atlántico de manera segura y consistente durante el siglo XVI. Esta empresa necesitó la creación de complejas redes tecnológicas e instituciones dirigidas a solucionar tal desafío, y a sostener el imperio y sus relaciones en los territorios de ultramar, dominando el mar con sus barcos.

Nieto nos invita a pensar el mundo ibero-atlántico del siglo XVI como un sistema de redes de actores (Latour, 2005; Law, Hassard, 1999) conformadas por humanos y no humanos puestas en marcha para la comprensión del Nuevo Mundo. Este se articuló como una forma de autoridad epistémica, la cual se manifestó en la cosmografía, la burocracia, la cartografía y la historia natural. A través de los manuales de navegación y los cronistas de indias, el autor nos presenta un período en el cual el contacto entre dos continentes reformuló el conocimiento europeo, poniendo en tensión la autoridad de autores clásicos como Plinio, Aristóteles y Ptolomeo, gracias a la experiencia de los pilotos y cosmógrafos que cruzaron el Atlántico.

El primer argumento del libro muestra que las tensiones con estas fuentes de autoridad permitieron la validación de la experiencia como una nueva forma de autoridad epistémica. La articulación de instituciones imperiales, manuales e instrumentos de navegación, cartografía y cronistas dieron pie a la consolidación y validación de una nueva forma de conocer y medir el mundo.

El cruce atlántico no solo necesitó de galeones capaces de resistir las duras condiciones de la navegación en alta mar: simultáneamente significó un despliegue técnico capaz de entrenar pilotos, mantener la legitimidad de la corona dentro de los barcos y en los territorios de ultramar, así como la actualización constante de mapas. A través de la Casa de Contratación de Sevilla y la figura del “piloto mayor”*, *el autor argumenta que el Estado imperial era una organización técnica y científica, ya que la ciencia y la tecnología son elementos de gobierno y control (Nieto, 2022, p.73). En este sentido, Sevilla jugó un papel determinante, ya que, a través de su localización geográfica y sus instituciones (la Casa de Contratación y el Consejo de Indias), centralizó la información que entraba y salía del puerto, tornándose el centro de control, cálculo y manejo de las operaciones marítimas y de exploración del Imperio. El puesto de piloto mayor (1508), no solo tuvo la constante necesidad de actualizar mapas y determinar las mejores rutas de navegación, sino también la demanda por entrenar a futuros pilotos a través de manuales de navegaciones y cátedras.

Cruzar el Atlántico en el siglo XVI necesitó del desarrollo de una nueva forma de navegar, una de altura – que permitiera salir del Mediterráneo, encontrar puntos de referencia en los astros y poder trazar rumbos – necesita de personas entrenadas y capaces de realizar mediciones en el medio del mar. Esto nos lleva al segundo argumento del libro, el problema y desafío de la estandarización. Problemas técnicos tales como medir la distancia que un barco ha recorrido, determinar la longitud de un mástil, o la hora del cambio de turno de la tripulación en un barco, demandaron la necesidad de estandarizar ciertas unidades de medida y centralizar la producción de madera, relojes de arena, astrolabios y agujas de marear. A través de la figura de “las maquinas del imperio” (Linebaugh, Rediker, 2005), el autor presenta a los barcos como redes en donde humanos y no humanos están en constante interacción (pilotos, maestres, marineros, pajes, instrumentos de navegación, el viento, el clima, Dios y demonios). El problema de estandarización, entonces, no solo se ve en el control sobre los instrumentos, barcos y burocracia que cruzaron el atlántico, también se puede observar en el orden y control de las tripulaciones de los barcos del imperio español.

El proyecto imperial español, nos propone el autor, es inseparable del proyecto de expansión religiosa. Siendo quizás la propuesta más innovadora de este libro, el autor afirma, en su tercer argumento, que la religión proporcionó una racionalidad y un sentido práctico para que los marineros se arriesgaran a cruzar el Atlántico. Por ende, la vida en los barcos estaba supeditada al calendario y las oraciones realizadas cada media hora, tras el giro de un reloj de arena que ordenaban la vida y rutina del barco. Así mismo, la religión explicaba fenómenos naturales como tormentas y naufragios, así como la dirección de la brújula. Todo en función de la labor de la divina providencia.

Finalmente, el cuarto argumento del texto nos lleva a pensar el papel que la cartografía y la historia natural tuvieron en el proceso de comprensión y apropiación del Nuevo Mundo. A través de las narraciones de Fernández de Oviedo, Francisco Hernández y Bernardino de Sahagún, el autor nos lleva a considerar como América se construyó y representó sobre marcos de referencia europeos como la biblia y las descripciones de Plinio y Aristóteles, y cómo la experiencia de los españoles para traducir y apropiar aquello que parecía exótico e inimaginable se tradujo en la formulación de una relación eurocéntrica que posicionó a Europa en el centro del mundo.

*Exploration, religion and Empire in the sixteenth-century Ibero-Atlantic world* es un libro que, además de ser meticuloso y estar bellamente ilustrado, propone repensar la tradición historiográfica de la revolución científica y el surgimiento de la modernidad, y pensar la cosmografía, cartografía, e historia natural española a partir de la empresa naval que cruzó el Atlántico, como uno de los antecedes más importantes dentro de la configuración de la ciencia moderna. Por otro lado, el texto nos permite reevaluar el rol de la ciencia dentro del proyecto imperial ibérico, incorporando nuevos elementos como los objetos, artefactos y agentes no humanos que hicieron parte de este proceso. El uso de fuentes primarias sugerentes y su estudio cuidadoso y disciplinado permiten aproximarse al siglo XVI desde el punto de vista de un problema técnico y humano, y desde el punto de vista de una historia global. En suma, el autor recrea una imagen sugestiva del siglo XVI en donde la construcción de conocimiento cartográfico, cosmográfico y natural fue influenciada por las necesidades de dominar a distancia y los retos que esto suponía, además de su profunda relación con la religión católica en la construcción temprana de la modernidad.
